# Demonstrating microbial co-occurrence pattern analyses within and between ecosystems

**DOI:** 10.3389/fmicb.2014.00358

**Published:** 2014-07-18

**Authors:** Ryan J. Williams, Adina Howe, Kirsten S. Hofmockel

**Affiliations:** ^1^Department of Ecology, Evolution, and Organismal Biology, Iowa State UniversityAmes, IA, USA; ^2^Mathematics and Computer Science, Argonne National LaboratoryArgonne, IL, USA; ^3^Microbiology and Microbial Genetics, Michigan State UniversityEast Lansing, MI, USA

**Keywords:** co-occurrence, microbial communities, network theory, community assembly, MGRAST

## Abstract

Co-occurrence patterns are used in ecology to explore interactions between organisms and environmental effects on coexistence within biological communities. Analysis of co-occurrence patterns among microbial communities has ranged from simple pairwise comparisons between all community members to direct hypothesis testing between focal species. However, co-occurrence patterns are rarely studied across multiple ecosystems or multiple scales of biological organization within the same study. Here we outline an approach to produce co-occurrence analyses that are focused at three different scales: co-occurrence patterns between ecosystems at the community scale, modules of co-occurring microorganisms within communities, and co-occurring pairs within modules that are nested within microbial communities. To demonstrate our co-occurrence analysis approach, we gathered publicly available 16S rRNA amplicon datasets to compare and contrast microbial co-occurrence at different taxonomic levels across different ecosystems. We found differences in community composition and co-occurrence that reflect environmental filtering at the community scale and consistent pairwise occurrences that may be used to infer ecological traits about poorly understood microbial taxa. However, we also found that conclusions derived from applying network statistics to microbial relationships can vary depending on the taxonomic level chosen and criteria used to build co-occurrence networks. We present our statistical analysis and code for public use in analysis of co-occurrence patterns across microbial communities.

## Introduction

Co-occurrence relationships are ecologically important patterns that reflect niche processes that drive coexistence and diversity maintenance within biological communities (Tilman, 1982; HilleRisLambers et al., 2012). In microbial systems, niche processes like environmental filtering where abiotic factors define specific habitat limits can support coexistence (Horner-Devine et al., 2007; Costello et al., 2009; Ofiţeru et al., 2010; Langenheder and Székely, 2011; Stegen et al., 2012), which are illustrated by co-occurrence patterns within communities. Species pairs or assemblages that co-occur may share similar ecological characteristics (Leibold and McPeek, 2006; Fuhrman and Steele, 2008; Raes and Bork, 2008; Chaffron et al., 2010; Eiler et al., 2012), which can be used to infer life-history strategies (Freilich et al., 2010; Barberán et al., 2012) and possibly to identify traits or even culture poorly understood microorganisms (Duran-Pinedo et al., 2011; Faust and Raes, 2012; Sun et al., 2013). Thus, applying co-occurrence analyses to microbial systems can provide valuable information for characterizing the biogeography, functional distribution or ecological interactions of microbes at the community scale or for identifying ecological traits of taxa that co-occur with well-characterized microorganisms.

Analyses of microbial co-occurrence patterns have been applied to a variety of research questions regarding biological interactions between organisms. Co-occurrence relationships have been useful in elucidating coexistence patterns spanning from pairs of microbial taxa in a range of ecosystems (Eiler et al., 2012; Kittelmann et al., 2013; Zhalnina et al., 2013) and functional groups (Duran-Pinedo et al., 2011; Bowen et al., 2013) to plant-microbe interactions (King et al., 2012). Classically, co-occurrence analysis has used checkerboard scores based on the presence or absence of organisms (Stone and Roberts, 1990), while larger datasets have been explored using correlation coefficients to represent either coexistence or competitive exclusion between two microbial taxa (e.g., Kittelmann et al., 2013). Subsequently, co-occurring pairs of microorganisms have been visualized using network methods (e.g., Fuhrman and Steele, 2008; Barberán et al., 2012) or ordination techniques [nonmetric multidimensional scaling (NMDS)] as seen in King et al. (2012). Though these visualization methods are useful, there are very few examples of applying network statistics to microbial co-occurrence despite their growing popularity among subfields of ecological and evolutionary research (Proulx et al., 2005). Network statistics can be used to determine the importance of microorganisms in co-occurrence networks (e.g., degree, betweenness, measures of centrality), possibly identifying keystone species within an ecosystem (Bauer et al., 2010; Steele et al., 2011; Eiler et al., 2012). Additionally, little effort has been made to identify a multivariate test for differences in microbial community co-occurrence patterns between ecosystems. Coupling co-occurrence patterns within microbial communities to network or multivariate methods can enhance interpretation and therefore increase knowledge related to microbial co-occurrence.

The integration of a variety of analyses that have been used to study microbial co-occurrence patterns can allow researchers to understand microbial coexistence at multiple levels of biological organization. For example, the use of bivariate regressions, network statistics, and multivariate tests can be used to understand microbial co-occurrence between microbial pairs, within groups of co-occurring microorganisms (e.g., modules), and whole communities, respectively. We developed an approach that integrates these methods and then used multiple datasets to demonstrate our approach. While many of these approaches have been used previously, our analytical framework integrates several methods and applies multivariate statistics to test for differences in co-occurrence across ecosystems. We have also tested the robustness of our framework by including multiple taxonomic levels and considering alternative criteria for the construction of co-occurrence networks. Our analysis was implemented to answer the following co-occurrence-related research questions: (1) Are co-occurrence patterns among microbial communities the same among ecosystems? (2) Within communities, are there distinct modules of co-occurring microorganisms, and are these consistent among ecosystems? (3) Are pairs of co-occurring microbes consistent among ecosystems, and can ecological traits be inferred from these relationships? (4) Do these co-occurrence relationship change at different taxonomic levels or with various criteria used to construct co-occurrence networks? To test this approach, we used three publicly available datasets from the Metagenomics Analysis Server (MGRAST; Meyer et al., 2008). We expected to find that the majority of co-occurrence relationships would differ strongly across ecosystems creating vastly different modules of interacting taxa within each ecosystem, while potentially a few relationships will exist between pairs of microorganisms as a reflection of biological interactions that are present independently of environmental factors.

## Materials and methods

**Figure 1:**
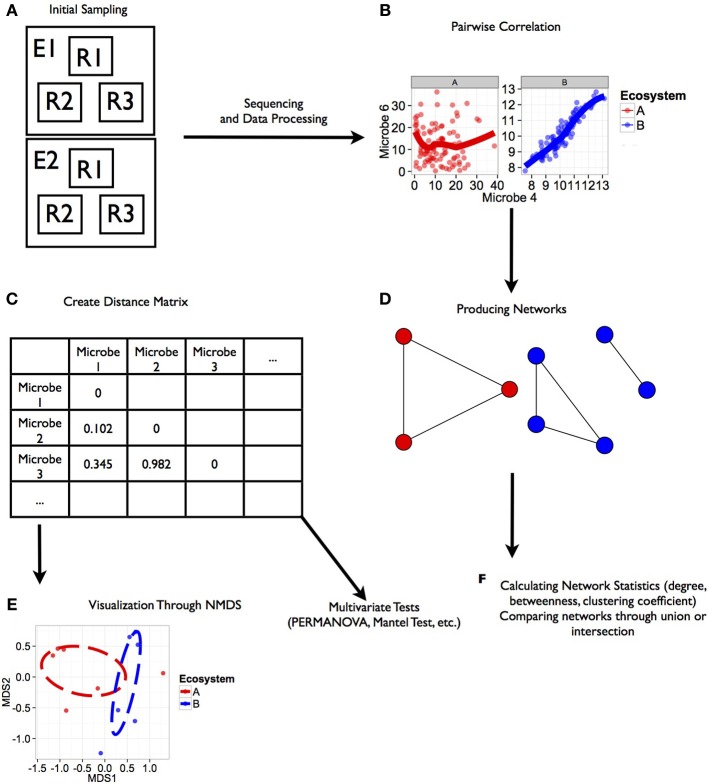
**Workflow for analysis of microbial co-occurrence between ecosystems.** This illustration represents a workflow from data collection through analysis stages for determining co-occurrence patterns among microbial communities. Each step in the workflow has been generated from simulated data. Scripts for the generating these figures are located in the Supplemental Material. **(A)** Ecosystems were sampled (E_1_, E_2_), and within each ecosystem several replicate groups of random samples were taken (R_1_, R_2_, R_3_). **(B)** Rank correlation represented by this regression plot was performed for two microbial orders (Microbe 4 and 6 shown here) within each environment that were consistent among replicate groups. **(C)** Distance matrices based on correlation coefficients between taxa were generated for downstream statistical tests. **(D)** Ecosystem-specific co-occurrence patterns were visualized using network diagrams. **(E)** Co-occurrence relationships between each ecosystem were visualized using NMDS. Further tests of network topology and distance matrices can be performed using a variety of multivariate tests like the mantel test or permutation multivariate analysis of variance (PERMANOVA). In the case of our simulated data, we found a significant effect of ecosystem on co-occurrence (PERMANOVA; *P* < 0.02). **(F)** Additional network statistics can be calculated to characterize networks, and networks can be compared to find shared relationships.

We designed a statistical approach written in R v. 3.0.1 (R Core Team, 2013). All scripts necessary to replicate this analysis are included in the Supplementary Material. The analysis presented in this paper is designed to test for differences in co-occurrence patterns at the community level across ecosystems, identify modules of co-occurring microorganisms within communities, and identify pairwise co-occurrence patterns within modules that are consistent across ecosystems (summarized in Figure 1). We considered co-occurrence to be positive rank correlations (Spearman's correlation) between pairs of microbes within each dataset with the strength of the relationship represented by the correlation coefficient (Figure 1B). Negative correlations (indicative of either competitive interactions or non-overlapping niches between microbes; Faust and Raes, 2012) were also included in this analysis though they were a small subset of our combined datasets. We only considered negative and positive co-occurrence relationships based on strength of correlation (i.e., ρ from the Spearman's correlation) at values less than or equal to −0.75 and −0.5 or greater than 0.5 and 0.75.

We applied our approach to determine co-occurrence patterns from three public datasets maintained through MGRAST that had replicated samples of 16S rRNA amplicon sequencing. Abundances of classified bacteria and archaea were accessed using the matR package (Braithwaite and Keegan, 2013), and were summarized at the order and family level with the assumption that microorganisms share similar traits at these phylogenetic levels. Though there is some evidence that certain traits are conserved at high levels of phylogeny (Philippot et al., 2010), we tested our analysis at multiple taxonomic levels as coherence of ecological patterns like co-occurrence may vary across different levels of taxonomy (Koeppel and Wu, 2012). The datasets were grouped following the schematic in Figure 1A with replicates nested within ecosystems. Ecosystems included apple flowers with and without antibiotic application [Shade et al., 2013b; 2 flower types (replicates) with 15 samples of each type], human body surfaces [Costello et al., 2009; 9 different bodies (replicates) divided into males and females with 24-25 samples each], and soils from different land-use types [Lauber et al., 2008; 5 different soils (replicates) with 4–43 samples each]. Datasets were chosen based on the number of replicates nested within similarly sampled ecosystems (i.e., flowers, body surfaces, or soils), and were classified generally into different replicates within each ecosystem. While the classification of these samples may not represent ideal replicates from each study [e.g., communities differ across body surfaces rather than sex or individual in the study by Costello et al. (2009) and communities did not differ across flower antibiotic treatments (Shade et al., 2013a)], they do provide enough statistical power to demonstrate our approach. Thus, it should be noted that biological interpretation of our results requires further exploration through controlled studies.

Before beginning our analysis, we rarefied samples to standardize for sequencing depth between samples. Prior to rarefication, samples ranged between 2 and 12,000 sequences per sample and a mean ranging from 1000 to 5000 depending on ecosystem type; these values were similar across taxonomic levels. We chose to use the minimum amount of counts per sample from the Shade et al. (2013b) datasets as this number was roughly the average for all samples used in our analysis. However, this rarefication step led us to using only two different soils from Lauber et al. dataset (2008) and three female body datasets from Costello et al. (2009). Though this rarefication step reduced the number of datasets used, it also removed less abundant taxa that can produce spurious co-occurrence relationships with highly abundant taxa (Faust and Raes, 2012). For the order dataset, samples were rarefied to 1407 reads per sample while the family dataset was rarefied to 1353 reads per sample.

### Testing for differences in co-occurrence patterns at the community level

To test for differences in co-occurrence patterns between microbial communities from different ecosystems, we generated a dissimilarity matrix consisting of Spearman correlation coefficient distances (1-correlation coefficient) representing co-occurrence between all pairs of microorganisms from each sample (Figure 1C) using the bioDist package (Ding et al., 2014). The calculation of these distances produces a matrix where microbial taxa rather than samples were compared to one another. This Spearman's distance matrix represents the strength of correlation among microbial pairs; thus smaller distances represent stronger correlations, which were visualized using non-metric multidimensional scaling (NMDS; Figure 1E). We used a permutational multivariate analysis of variance (PERMANOVA; 9999 permutations) (Anderson, 2001) from the vegan package (Oksanen et al., 2013), with ecosystem type (apple flower, bodies, or soils) representing our independent variable to test for differences in co-occurrence patterns at the community level based on the Spearman's distance matrix.

The generation of this Spearman's dissimilarity matrix and its use in a PERMANOVA has not been described previously to our knowledge; therefore we generated simulations under a variety of conditions that represent null cases and significant differences in community co-occurrence patterns between ecosystems (R script in Supplementary Material). The null case represents a situation where correlations between two microorganisms within a community are no greater than any correlation with a microorganism sampled from another ecosystem, where no correlation is expected. If correlations between microorganisms within a community were strong and consistent across replicates from the same ecosystem, this null hypothesis would be rejected (Supplementary Figure 1).

### Delineating modules of co-occurring microorganisms and consistent co-occurrence relationships

We illustrated modules of co-occurring microorganisms within communities where microbial taxa represent nodes and the presence of a co-occurrence relationship based on correlation is represented by an edge (Figure 1D). These correlation relationships were generated for each pair of microbial taxa within each ecosystem replicate as long as both taxa had abundance greater than 0. We made a consensus network of co-occurrence relationships within each ecosystem based on the strength of the correlation (ρ from the Spearman's correlation), and co-occurrence relationships were only included if they occurred across all ecosystem replicates. Though this method has been illustrated to produce some spurious co-occurrence relationships among simulated data (Friedman and Alm, 2012), this rank-based correlation statistic does not require any transformation of variables to fit assumptions of normality and may outperform Pearson's correlations. To increase our level of stringency that may reduce the appearance of spurious co-occurrences within our networks, pairwise relationships had to be consistent across all datasets of a given ecosystem type, greatly reducing the number of co-occurrence pairs (Chaffron et al., 2010).

Networks were produced using the igraph package (Csardi and Nepusz, 2006) where each network was the union of positive co-occurrences or negative co-occurrences (less than −0.5 or greater than 0.5) that were consistent within each ecosystem. Unconnected nodes were removed along with loops that indicate microbial taxa were correlated with themselves using the “delete.vertices” and “simplify” functions, respectively. We performed this through the “graph.union.by.name” function from the igraph package. Modules were designated as groups of highly connected microbes (modules) that were poorly connected to others. Modules were detected using an algorithm based on edge betweenness through the “edge.betweenness.community” function in igraph (Girvan and Newman, 2002; Newman and Girvan, 2004). The method used in our analysis looks for edges (i.e., co-occurrence) that are the most between vertices (microbes), and thus finding edges that are responsible for connecting many other microbial groups (Girvan and Newman, 2002). This method differs from agglomerative methods [e.g., measures of “cliquishness” (Watts and Strogatz, 1998)], which have been demonstrated in protein-network clustering (Bader and Hogue, 2003; Rivera et al., 2010). Instead, the betweenness centrality method we use is designed for simple graphs with single-type vertices as opposed to bipartite graphs, and avoids hierarchical clustering issues that can occur with agglomerative methods (Girvan and Newman, 2002). We also looked for intersections between networks from different ecosystems using the “graph.intersection.by.name” function (igraph) to determine if any co-occurrence relationships were consistent across ecosystems.

## Additional statistical analyses

To characterize differences in community composition between ecosystems, we performed a PERMANOVA with Bray–Curtis dissimilarity on our initial community matrices (for both microbial orders and families) with abundances scaled between 0 and 1. This analysis was performed using the “decostand” and “adonis” functions from the vegan package in R (Oksanen et al., 2013). We generated nonmetric multidimensional scaling (NMDS) plots to visualize differences in community composition using Bray–Curtis dissimilarity as well.

We were also interested in generating statistics that describe the network that may be important for understanding co-occurrence relationships. We produced network statistics that describe the position and connectedness of microorganisms within each co-occurrence network. This included normalized node degree, which is the number of co-occurrence relationships that a microorganism is involved in a network normalized by the total number of nodes using the “degree” function (igraph package; Csardi and Nepusz, 2006). We also calculated betweenness scores for each microbial taxonomic group using the “betweenness” function from igraph (Csardi and Nepusz, 2006), which is defined by the number of paths through a focal microbial node. Additionally, we calculated clustering coefficients using the “transitivity” function for comparison to other networks as performed in Steele et al. (2011).

We then determined relationships between degree and betweenness. Initial visualization of relationships betweenness and degree appeared to be correlated and non-linear. Thus we fit mixed models within each ecosystem and each level of correlation strength with degree as an independent variable, betweenness as a response variable, and ecosystem replicate as a random factor based on a power function (αx^β^). Mixed models were fit using the lme4 package in R (Bates et al., 2014). With this analysis we hoped to identify microbial taxa that are highly connected that may represent keystones within their ecosystem (Steele et al., 2011; Faust and Raes, 2012). We expanded this concept of keystone species to include both degree and betweenness, as these metrics illustrate both the number of connections and how important those connections are to the overall network. Therefore, we identified keystone taxa as those with the highest predicted betweenness based on our mixed models.

## Results

### Differences in co-occurrence patterns at the community level

We first quantified differences in community composition and community co-occurrence across ecosystems using a PERMANOVA and the Bray–Curtis dissimilarity and Spearman's distance, respectively. Although differences in community composition were clear among microbial orders and families (Supplemental Figure 1, *P* < 0.0001 for both), no clear difference was seen in co-occurrence patterns (*P* > 0.05). The lack of differences was clear in the visualization through NMDS as samples from each ecosystem completely overlapped one another (data not shown). The lack of differences in community co-occurrence patterns were likely driven by weak or non-significant correlations between most taxa within each ecosystem (see Supplementary Material for simulation of this case). Thus, our approach did not detect differences between co-occurrence patterns between samples from different ecosystems. In other words, the majority of microorganisms within a single ecosystem replicate were uncorrelated, and therefore equally uncorrelated to microorganisms from any other ecosystem replicate. If stronger correlations existed within a single ecosystem replicate as compared to other unrelated replicates, the explanatory power of this analysis would increase (see Supplementary Material).

### Delineating co-occurring modules and pairs

**Figure 2:**
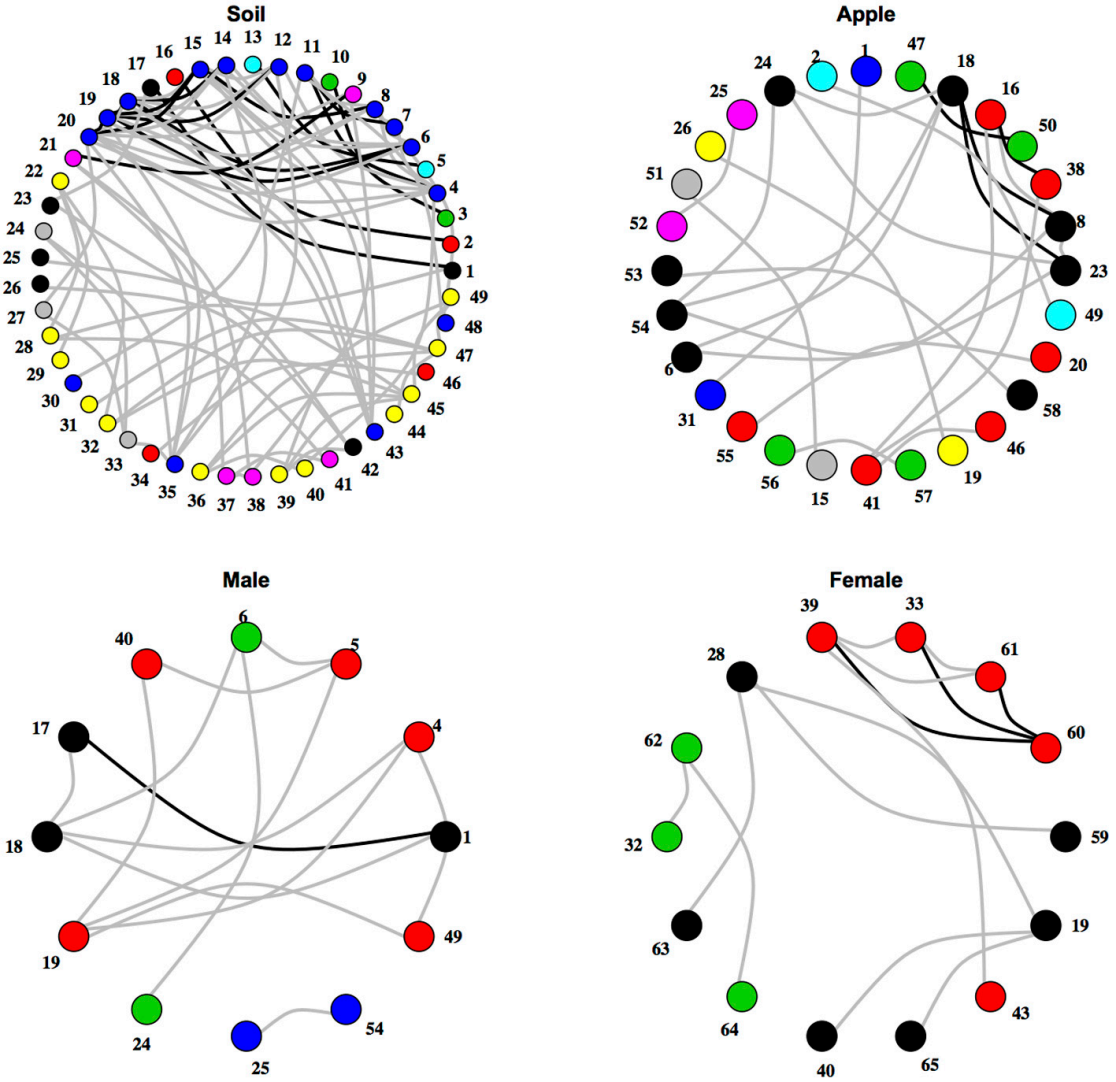
**Networks of co-occurring microbial orders within ecosystems.** Networks represent relationships between co-occurring ecosystems. Edges colored in black represent co-occurrence relationships that were consistent at the 0.75 correlation level, while edges in gray represent co-occurrence relationships that were consistent at the 0.5 correlation level. Numbers represent microbial orders seen in Supplementary Table 6. Node color represents module membership.

After testing for differences in community co-occurrence patterns between ecosystems, we aimed to identify consistent groups or modules of co-occurring microbial taxa among replicate samples within an ecosystem (Figure 2; Supplementary Tables 2, 3). When considering microbial orders, the apple ecosystem had the most modules at 11 followed by male samples with 4 and female and soil both with 3. When classifying microbial families into modules, a different trend was found. Soil had the most modules at 18, followed by apple at 14, female with 7, and male with 5. Negative co-occurrence modules were not found in any of the body samples (male or female), while soil had the most (9 order modules, 7 family modules) and apple had only a few (3 order, 4 family). In general, modules contained between either 2–6 orders or families, and each ecosystem usually had one large module containing multiple taxa. For example among soil families, one module contained 41 taxa while other soil family modules contained between 2 and 10 taxa. Modules were often found to be composed of multiple unrelated bacterial orders or families that were not necessarily associated at higher taxonomic levels. Thus, module delineation did not necessarily follow phylogenetic relationships among microbial communities categorized at the level of orders or families.

We then aimed to determine pairwise co-occurrence relationships that were consistent across ecosystems through the intersection of networks from different ecosystems (Table 1). Overall, more microbial families co-occurred across ecosystems than microbial orders, and no co-occurrence relationships held across all ecosystems. Also, relationships found at one taxonomic level were not necessarily found at another level. For example, Cytophagales and Flavobacteriales co-occurred across soil and apple ecosystems, and this relationship held true between Cytophagaceae and Flavobacteriaceae. Alternatively, Micrococcaceae from the Actinomycetales and Nitrosomonadaceae from the Nitrosomonadales co-occurred at the family level, but their respective orders did not co-occur. Furthermore, important co-occurrence relationships among families within the same order, such as Micrococcaceae and Microbacteriaceae from the Actinomycetales, were not detectable when considering microbial order alone.

**Table 1:** **Pairwise co-occurrence relationship statistics**.

**Ecosystem comparison**	**Orders**	**Families**
Soil—Apple	Cytophagales—Flavobacteriales	Clostridiaceae—Mycobacteriaceae
	Cytophagales—Sphingobacteriales	Cytophagaceae—Flavobacteriaceae
		Cytophagaceae—Oxalobacteraceae
		Cytophagaceae—Propionibacteriaceae
		Cytophagaceae—Sphingobacteriaceae
		Microbacteriaceae—Micrococcaceae
		Microbacteriaceae—Propionibacteriaceae
		Microbacteriaceae—Pseudonocardiaceae
		Microbacteriaceae—Sphingobacteriaceae
		Micrococcaceae—Nitrosomonadaceae
		Micrococcaceae—Propionibacteriaceae
		Micromonosporaceae—Promicromonosporaceae
		Propionibacteriaceae—Pseudonocardiaceae
		Propionibacteriaceae—Sphingobacteriaceae
		Rhodocyclaceae—Rhodothermaceae
Soil—Male	Acidimicrobiales—Solirubrobacterales	Acidimicrobiaceae—Conexibacteraceae
	Burkholderiales—Sphingobacteriales	Cytophagaceae—Nocardioidaceae
	Cytophagales—Sphingobacteriales	Microbacteriaceae—Oxalobacteraceae
		Oxalobacteraceae—Rhodobacteraceae
Soil—Female		Microbacteriaceae—Propionibacteriaceae
		Microbacteriaceae—Sphingomonadaceae
Apple—Male		Cytophagales—Sphingobacteriales
		Propionibacteriaceae—Sphingomonadaceae
Apple—Female		Clostridiales Fam. XI *Incertae Sedis*—Corynebacteriaceae
		Microbacteriaceae—Propionibacteriaceae
Male—Female	Pseudomonadales—Sphingomonadales	Corynebacteriaceae—Mycobacteriaceae
		Moraxellaceae—Pseudonocardiaceae
		Moraxellaceae—Sphingomonadaceae

Pairs of microbial taxa represent consistent positive pairwise relationships across the designated ecosystems.

### Co-occurrence network statistics

**Figure 3:**
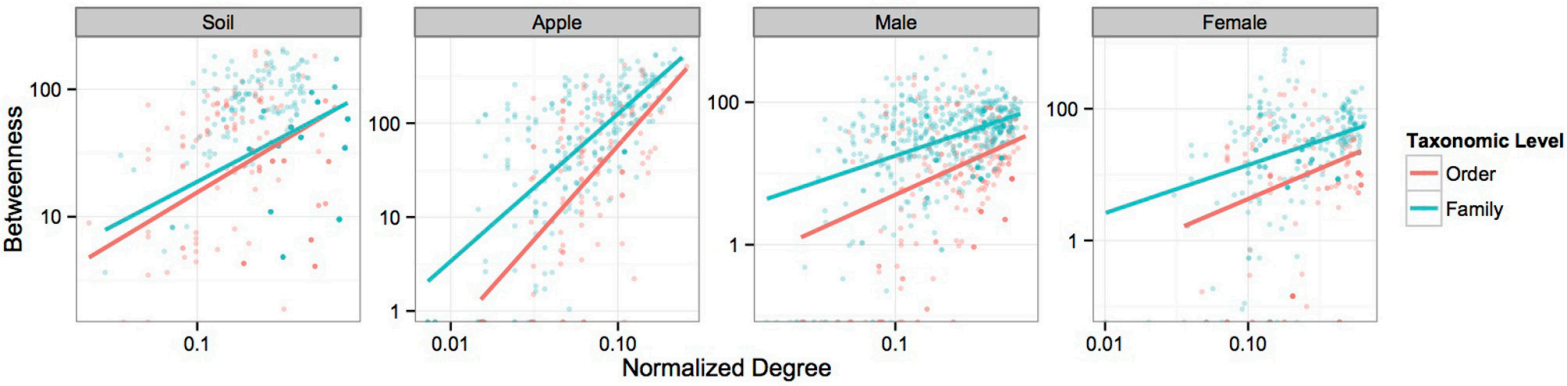
**Power-function relationships between node degree and betweenness.** Figures represent the power-function relationships between node degree and betweenness for microbial orders and families within each ecosystem at the 0.5 correlation level. Scales are log transformed. Each best-fit line represents the predicted values seen in Supplementary Table 4 for each correlation cutoff.

We first visualized networks within each ecosystem for both positive and negative co-occurrence relationships (Figure 2, Supplemental Figure 2). We then calculated a normalized degree and betweenness score for nodes within each network and modeled relationships between these variables as a power function, αx^β^, using mixed models. The slopes of each power function within an ecosystem were similar across taxonomic levels when considering correlations greater than 0.05 (Figure 3). However, when considering more stringent correlation cutoffs, greater disparity was seen across power functions within an ecosystem (Supplementary Figure 3), suggesting that the choice of taxonomic level or correlation strength may have a significant effect on the interpretation of co-occurrence networks. All but two cases had significant slope parameters (β; Supplementary Table 4), and involved correlation cut offs of either 0.75 or −0.75. When considering the slopes across different strengths of correlation, models based on negative co-occurrence networks often produced higher values of β; this was especially true when considering correlations less than or equal to −0.5. We also calculated clustering coefficients for comparison to other biological networks. We found that while all networks fell across a range of values common to other networks (Steele et al., 2011), only positive co-occurrence networks displayed “small-world” characteristics (Watts and Strogatz, 1998), where nodes were more connected on average than may be expected at random (Supplemental Table 5).

We then used the predictions of our mixed models to determine keystone taxa within each network of positive co-occurrences. When considering networks with different correlation cutoffs within a similar ecosystem, the top keystone taxa were not necessarily the same. In soils, Bacillales, Actinomycetales, and Clostridiales were the top keystone orders with a cutoff of 0.75 while Thermoleophilales, Desulfovibrionales, and Sphingobacteriales were designated as the top keystone orders with a cutoff of 0.5. However when moving down to the family level in soils, keystone taxa were much more consistent between networks with different correlation cutoffs. These results suggest that applying ecological characteristics to network elements must happen under careful consideration of the parameters used to delineate co-occurrence relationships.

## Discussion

The exploration of co-occurrence networks is a useful method for determining biological interactions occurring within microbial communities. Here we have laid out a framework to generate co-occurrence networks and to compare co-occurrence relationships within and between ecosystems. A novel strength of this framework is its utility at multiple scales; analysis can be performed to observe co-occurrence from the community level down to pairwise interactions between microbial taxa. We applied our analyses to three datasets to demonstrate its effectiveness and determine differences in co-occurrence between ecosystems. Through this investigation, we were able to distinguish co-occurring pairs of microbial orders and families that were consistent across ostensibly different ecosystems, while the majority of co-occurrence relationships within ecosystems appear to be at random (i.e., uncorrelated microbial pairs). Additionally, we were able to distinguish modules of co-occurring microorganisms that appear to behave similarly within communities. These results and our approach can be used to explore microbial communities in a variety of ecological contexts including but not limited to the identification of biotic and abiotic drivers of microbial community assembly, identification of keystone microbial species, or inferring ecological characteristics of poorly understood or unculturable microbial taxa.

The analytical framework that we present has been able to detect ecologically relevant relationships between microbial taxa. For example, we were able to detect consistent positive co-occurrence between two skin-dwelling bacteria, Pseudomonadales and Sphingomonadales, across male and female body datasets. One important use of our analytical framework is the development of hypotheses regarding traits of rarely studied microbes through co-occurrence with other microorganisms based on the assumption that coexisting species are ecologically similar (Leibold and McPeek, 2006; Barberán et al., 2012). For example, the recently described order, Solirubrobacterales, has been noted to occur in soils with little information regarding its ecological role (Shange et al., 2012). Our co-occurrence analysis suggests that Solirubrobacterales either assumes analogous ecological roles or is selected by similar environmental factors as its co-occurring taxa in soil (Figure 2). Strains from Acidomicrobiales and Actinomycetales are known to overlap in their carbon substrate use (Goldfarb et al., 2011). These results illustrate potential resource utilization roles that minimize interspecific competition through niche partitioning, where Solirubrobacterales can coexist with Acidomicrobiales and Actinomycetales by utilizing alternative substrates. Alternatively, these three heterotrophic orders may have overlapping carbon substrate preference, yet competition between the three orders is minimized under C-rich soil conditions. Indeed, the relationship between Solirubrobacterales and Acidomicrobiales and the related families, Acidimicrobiaceae and Conexibacteraceae, may be ecologically relevant as these co-occurrence relationships occurred in both soil and male body ecosystems (Table 1). The relationships between these groups of microorganisms represent testable hypotheses regarding coexistence between newly described bacteria like the Solirubrobacterales and other microbial heterotrophs. Furthermore, hypotheses can address higher levels of hierarchical organization among co-occurring pairs by exploring relationships between microbial taxa with similar life history (e.g., heterotrophy) that exist within the same module indicating similar niches (Chow et al., 2013). All together, these relationships represent potential hypotheses driven by analysis through our co-occurrence approach and require the inclusion of more replicated microbial community data to confirm coexistence between these microbial taxa.

In microbial systems, much attention has been paid to the deterministic or stochastic assembly of communities. While stochastic processes may play a partial role in microbial community assembly, environmental filtering or selection by abiotic factors can be important in both experimental (Ofiţeru et al., 2010; Langenheder and Székely, 2011; Faust and Raes, 2012) and naturally occurring communities (Horner-Devine et al., 2007; Costello et al., 2009; Stegen et al., 2012). We used our analysis framework to test for differences in co-occurrence networks at the community level, and found that though community composition strongly differs between ecosystems (Figure 2), no significant differences existed among community co-occurrence. Rather, few co-occurrence relationships are strong within ecosystems, yet some of the co-occurrences are consistent across ostensibly different ecosystems. These results suggest that environmental filtering plays a strong role in driving microbial community composition and fluctuations among microbial populations are generally independent of one another. However, further examination of uncorrelated microbial populations across more ecosystems is necessary, as these datasets were not collected to explicitly test microbial co-occurrence and the scale at which samples were collected may not be relevant for microbial community interactions. It has been suggested that some microbial taxa may be more affected by biotic factors, while others are more affected by abiotic factors (Fuhrman and Steele, 2008), which may create complex patterns within co-occurrence networks that we could not detect with this method. Though our analysis was able to illustrate differences in co-occurrence at the community level among simulated data (Supplemental Material), the true data used in our analysis was much more complex and had less replications. Further application of PERMANOVA for co-occurrence may need to consider the amount of replication necessary to pick up differences in community co-occurrence among “noisy” natural data. Also, the incorporation of continuous environmental covariates may explain variation in co-occurrence or determine at least the abiotic effects on community co-occurrence (Steele et al., 2011) as it has already been used to forecast microbial community composition (Larsen et al., 2012). Nevertheless, these results indicate that the majority of biological interactions between microbial taxa are ecosystem dependent much like microbial temporal dynamics (Shade et al., 2013a), and consistent biological interactions among microorganisms may be a special case rather than the norm when considering microbial communities as a whole and at high taxonomic levels.

Though we were able to demonstrate the usability of our analytical framework and find potentially useful interactions between microbial taxa, there are a few shortcomings to what we present here. One aspect of our analysis that we did not test is the relative contribution specific ecosystem replicates may have on overall co-occurrence relationships. Unequal sample sizes among replicates is an experimental factor worth considering as the use of PERMANOVA and other multivariate tests can be sensitive to unbalanced designs (Anderson and Walsh, 2013). Also, the number of ecosystem replicates might affect our ability to detect consistent co-occurrence patterns. Our apple and soil datasets had two ecosystem replicates, while the body dataset consisted of three and six replicates for female and male bodies, respectively. With greater replication across all ecosystems, one might relax their criteria for determining consistent co-occurrence relationships and instead consider the distribution of correlation coefficients across replicates. Additionally, special consideration may be needed when choosing module-detection algorithms, and comparisons between agglomerative (Watts and Strogatz, 1998; Rivera et al., 2010) and divisive methods as we used here (Girvan and Newman, 2002). Though it should be noted that the networks we analyzed were fairly simple and may not vary largely depending on the community detection method.

We also chose in our analysis to assemble networks based on correlation coefficients without consideration of the involved *p*-value. When considering correlation strength cutoffs, we produced different networks (Figure 2), and statistics like degree and betweenness calculated from these models were different as well. Therefore biological interpretation of these statistics may need to consider the sensitivity of these biological interpretations to changes in criteria determining network relationships. Similarly, we did test whether cut-offs based on *p*-values, or adjusted *p*-values based on false discovery rate (*q*-value; Strimmer, 2008) affected our results (data not shown). We observed that an adjustment based on false discovery rate actually produced *q*-values less than *p*-values based on pairwise correlations (Pike, 2011). It is important to note that each ecosystem and the datasets belonging to each ecosystem had varying samples size, which can also affect the *p*-value of the correlation. Despite differences in sample sizes among these data, and the variety of methods that exist today in analyzing networks, the results we have presented are a clear and accessible example of how our analytical framework for co-occurrence analysis allows for deep investigation of environmental factors and biological interactions occurring at multiple scales of biological organization. Co-occurrence relationships found in our study necessitate further observation across multiple datasets and empirical tests that determine the mechanisms driving co-occurrence between specific microorganisms.

The use of network algorithms and statistics to understand co-occurrence within communities can play an important role in understanding drivers of community assembly among microorganisms (Faust and Raes, 2012). Expanding previous research that focuses on bivariate comparisons of microbial taxa (e.g., Zhalnina et al., 2013) through the use of multivariate techniques as we have demonstrated here is an important next step. The statistical analyses that we provide can be applied to any sort of community abundance data, and is not necessarily limited to microbial applications. Additionally, alternative measures of co-occurrence like sparCC (Friedman and Alm, 2012), maximal information coefficient (MIC; Reshef et al., 2011) may be incorporated throughout the framework instead of Spearman's correlation. When moving to lower levels of taxonomic resolution like species, it may be important to incorporate measures like MIC which has been demonstrated to identify relationships with fine taxonomic resolution (Reshef et al., 2011). However, the actual biological interpretation at this scale may be difficult, even when utilizing methods like MIC due to the number of co-occurrence relationships and the paucity of ecological data regarding the majority of 16S rRNA sequences. Our analysis does not strictly require the use of Spearman's correlations, and other methods that measure the strength of a relationship between pairs of microbes can be easily incorporated. Additionally, the Spearman's distance may be changed by scaling any other measure (MIC, for example) between 0 and 1, subtracting that value from 1, and thereby creating a distance matrix that can be incorporated into a multivariate framework.

Despite some of the shortcomings presented here, the framework we present may also be useful in conjunction with other methods that measure phylogenetic dispersion while investigating community assembly (Walter and Ley, 2011), and are easily calculated using phylogenetic trees used or created in through sequencing pipelines (e.g., QIIME; Caporaso et al., 2010). Additionally, using genomic data that relate traits across wide spans of phylogeny (e.g., Zimmerman et al., 2013) or the combination of metagenomic data and phylogenetic relationships (e.g., Segata et al., 2012), may be used to validate ecological inferences based on co-occurrence. Linking these traits with modules of co-occurring microorganisms may be useful for identifying functional groups within communities, where modules rather than individual taxa may be used to simplify high-dimensional datasets. Furthermore, linking co-occurrence relationships with both traits and environmental metadata (Fuhrman, 2009; Steele et al., 2011; Gilbert et al., 2012) may be applied in our framework to test for effects of abiotic factors on multiple levels of co-occurrence. The calculations of additional network statistics can be performed at the node, edge, or network level like clustering coefficients (Steele et al., 2011), which can easily be incorporated into scripts included in the Supplementary Material. Though the applicability of our approach is broad, the results we present here a demonstration of our analytical framework and are also hypotheses meant for further investigation. Further application of co-occurrence analysis is necessary in reduced experimental systems to conclude that co-occurrence relationships found here are driven by biological or environmental factors (Gilbert et al., 2012), which in turn has proven successful in understanding uncultured microorganisms (Duran-Pinedo et al., 2011; Faust and Raes, 2012). Our co-occurrence framework represents a step toward understanding microbial ecology beyond community composition alone, and our analysis at multiple scales of biological organization can help us understand community assembly and coexistence among microorganisms (Raes and Bork, 2008; Fuhrman, 2009; Faust and Raes, 2012).

### Conflict of interest statement

The authors declare that the research was conducted in the absence of any commercial or financial relationships that could be construed as a potential conflict of interest.
