# Supplementary material for: Demonstrating microbial co-occurrence pattern analyses within and between ecosystems
Source: Front Microbiol. 2014 Jul 18;5:358. doi: 10.3389/fmicb.2014.00358 (PMC4102878; doi:10.3389/fmicb.2014.00358)
Supplement: Supplementary file 1 [file Presentation1.PDF]

Supplementary Material

Demonstrating Microbial Co-occurrence Pattern Analyses Within and Between Ecosystems

Ryan J. Williams[1], Adina Howe[2,3], Kirsten S. Hofmockel[1]*

[1]Department of Ecology, Evolution, and Organismal Biology, Iowa State University, Ames, Iowa, 50011, USA,
[2]Mathematics and Computer Science, Argonne National Laboratory, Argonne, Illinois, 60439, USA,[3]Microbiology and Microbial Genetics, Michigan State University, East Lansing, MI 48823, USA

* **Correspondence:** Kirsten S. Hofmockel, Iowa State University, Department of Ecology, Evolution, and Organismal Biology, 251 Bessey Hall, Ames, Iowa, 50011, USA
khof@iastate.edu

1. Supplementary Data

1.1 Simulating co-occurrence conditions and PERMANOVA

In the manuscript, Figure 1 is a graphical representation of our analytical workflow. In the script *co-occurrrence_permanova_sim.R*, all figures from can be created from simulated data. This represents an example of our workflow with 6 microbial taxa where the first 3 are correlated in ecosystem A, and the second 3 are correlated in ecosystem B.

To demonstrate both null and significant cases of our multivariate test for co-occurrence between ecosystems, we simulated microbial community data under a range of co-occurrence (correlation) strength. The R code for this simulation is in the second half of co-*occurrrence_permanova_sim.R.* The simulated data consisted of two ecosystems (A and B) each with 6 species. In ecosystem A, species 1-5 co-occur (i.e. correlated) and in ecosystem B, species 6-10 co-occur, meaning they are correlated as well. Species are simulated by random draws from a normal distribution in ecosystem A and B respectively. We altered the strength of co-occurrence relationships (correlation coefficient) by 0.01 between each co-occuring taxa from 1.0 to 0. At each correlation coefficient, we simulated our community 100 times and ran a PERMANOVA with 9999 permutations as described in the **Materials and Methods** section (data not shown). We noticed a positive relationship between strength of co-occurrence and the test's R^2 value. This increase in co-occurrence strength was also met with an exponential decay in model P-value. Model R^2 and P-value were negatively related, though there were large ranges in R^2 when P-values were less than 0.05. Nonetheless, these simulation results support the use of a PERMANOVA on a Spearman's distance matrix to test for differences between co-occurrence patterns. Though it may be useful to simulate PERMANOVAs with Spearman's distance matrices under a greater range of conditions, our results support the use of this test in conjunction with analyzing microbial co-occurrence patterns at the community scale.

1.2 R Scripts used to run co-occurrence analysis

All scripts used in this analysis along with data can be found at the following website: https://github.com/ryanjw/co-occurrence. These scripts are designed to be used from the R GUI program but can be edited to work through Shell scripting if needed. The datasets can be found in the folder labeled 'data'. This includes two comma-delimited files labeled *total_order_info.csv* and *total_family_info.csv*. Sample information was taken for each MG-RAST id and included in the file. Based on information given within metadata belonging to each sample, we created a general label describing it (e.g. soil, forest soil, individual, or the application of an antibiotic).

The first script used in our analysis is named *co_occurrence_pairwise_routine.R*. The input files can be either of the files located in the data folder, *total_order_info.csv* or *total_family_info.csv.* Rarefication is designated as 1407 for orders and 1353 for families, which must be changed by hand in the script. The input files are organized to have a column of metadata referring to the dataset that samples originate from. In the script, these are assigned to the vector 'trt'. The loop within the script uses this vector to iterate through each dataset. If the script is used for data other than what is provided, the *for* loop that has the iterator '*b*' must start at the first column containing abundance data (rather than metadata). The output is a data frame with a column for the dataset label, each microbial pair, the correlation coefficient, p-value, and the abundances of each microbe in the pair. The script labeled *permanova_script.R* will reorganize the initial dataset provided in the data folder and run a permutation multivariate analysis of variance (PERMANOVA). The output from this script is the general PERMANOVA output. It should be noted that this script can take a while to run.

The second script is labeled *edgelist_creation.R*. This produces the list of co-occurrence relationships in a format that can be used with igraph to create networks. The input file contains the results from the script, *co_occurrence_pairwise_routine.R.* Otherwise it requires a data frame consisting of columns that specify the dataset ('trt' as above), a column denoting each microbe in a pairwise relationship, and a column of values that is used to denote the strength of co-occurrence (in our case, rho, or Spearman's correlation coefficient). The output is a data frame with all edges in the network, p-values, correlation cutoff value, dataset label, and q-value from a false discovery rate correction.

The third script is labeled *network_statistics.R*. The input file contains the results from the script, *co_occurrence_pairwise_routine.R.* Like the script, *edgelist_creation.R*, it requires a data frame consisting of columns that specify the dataset, columns denoting microbes in pairwise relationships, and a column of values that is used to denote the strength of co-occurrence (in our case, rho, or Spearman's correlation coefficient). The output is a data frame including the specific microbe (order or family), correlation cutoff, dataset label, normalized degree, betweenness, clustering coefficient, clustering coefficient from a random network, and the ratio of these two coefficients.

The fourth script is labeled *comm_stat_function.R*. The input file is the results from the script, *co_occurrence_pairwise_routine.R.* Like the script, *edgelist_creation.R*, it requires a data frame consisting of columns that specify the dataset, columns denoting microbes in pairwise relationships, and a column of values that is used to denote the strength of co-occurrence (in our case, rho, or Spearman's correlation coefficient). The output from this script is a data frame of microbes, the module they belong to (a number), correlation cutoff, and dataset label.

2. Supplementary Figures and Tables

2.1 Tables

Supplemental Table 2. Co-occurrence modules at the order level.

Ecosystem	Order	Module	Class	Phlyum
Apple	Actinomycetales	1	Actinobacteria	Actinobacteria
Apple	Bacillales	1	Bacilli	Firmicutes
Apple	Clostridiales	1	Clostridia	Firmicutes
Apple	Cytophagales	1	Cytophagia	Bacteroidetes
Apple	Flavobacteriales	1	Flavobacteria	Bacteroidetes
Apple	Sphingobacteriales	1	Sphingobacteria	Bacteroidetes
Apple	Nitrosomonadales	2	Beta Proteobacteria	Proteobacteria
Apple	Rhodocyclales	2	Beta Proteobacteria	Proteobacteria
Apple	Solibacterales	2	Solibacteres	Acidobacteria
Apple	Thermoleophilales	2	Actinobacteria	Actinobacteria
Apple	Puniceicoccales	3	Opitutae	Verrucomicrobia
Apple	Verrucomicrobiales	3	Verrucomicrobiae	Verrucomicrobia
Apple	Acidimicrobiales	4	Actinobacteria	Actinobacteria
Apple	Desulfuromonadales	4	Delta Proteobacteria	Proteobacteria
Apple	Acidobacteriales	5	Acidobacteria	Acidobacteria
Apple	Xanthomonadales	5	Gamma Proteobacteria	Proteobacteria
Apple	Bacteroidales	6	Bacteroidetes	Bacteroidetes
Apple	Chlorobiales	6	Chlorobea	Chlorobi
Apple	Bacteroidetes Order II Incertae sedis	7	Bacteroidetes	Bacteroidetes
Apple	Sphingomonadales	7	Alpha Proteobacteria	Proteobacteria
Apple	Chlamydiales	8	Chlamydiae	Chlamydiae
Apple	Rhodobacterales	8	Alpha Proteobacteria	Proteobacteria
Apple	Chloroflexales	9	Chloroflexi	Chloroflexi
Apple	Thermomicrobiales	9	Thermomicrobia	Chloroflexi
Apple	Ktedonobacterales	10	Ktedonobacteria	Chloroflexi
Apple	Thiotrichales	10	Gamma Proteobacteria	Proteobacteria
Apple	Lactobacillales	11	Bacilli	Firmicutes
Apple	Rhodospirillales	11	Alpha Proteobacteria	Proteobacteria
Female	Bifidobacteriales	1	Actinobacteria	Actinobacteria
Female	Chromatiales	1	Gamma Proteobacteria	Proteobacteria

Female	Flavobacteriales	1	Flavobacteria	Bacteroidetes
Female	Pseudomonadales	1	Gamma Proteobacteria	Proteobacteria
Female	Rhizobiales	1	Alpha Proteobacteria	Proteobacteria
Female	Sphingomonadales	1	Alpha Proteobacteria	Proteobacteria
Female	Campylobacterales	2	Epsilon Proteobacteria	Proteobacteria
Female	Coriobacteriales	2	Actinobacteria	Actinobacteria
Female	Fusobacteriales	2	Fusobacteria	Fusobacteria
Female	Pasteurellales	2	Gamma Proteobacteria	Proteobacteria
Female	Selenomonadales	2	Negativicutes	Firmicutes
Female	Cytophagales	3	Cytophagia	Bacteroidetes
Female	Enterobacteriales	3	Gamma Proteobacteria	Proteobacteria
Female	Oscillatoriales	3	Cyanophyceae	Cyanobacteria
Male	Acidimicrobiales	1	Actinobacteria	Actinobacteria
Male	Solirubrobacterales	1	Actinobacteria	Actinobacteria
Male	Sphingobacteriales	1	Sphingobacteria	Bacteroidetes
Male	Burkholderiales	2	Beta Proteobacteria	Proteobacteria
Male	Caulobacterales	2	Alpha Proteobacteria	Proteobacteria
Male	Pseudomonadales	2	Gamma Proteobacteria	Proteobacteria
Male	Sphingomonadales	2	Alpha Proteobacteria	Proteobacteria
Male	Xanthomonadales	2	Gamma Proteobacteria	Proteobacteria
Male	Bacillales	3	Bacilli	Firmicutes
Male	Cytophagales	3	Cytophagia	Bacteroidetes
Male	Bacteroidales	4	Bacteroidetes	Bacteroidetes
Male	Clostridiales	4	Clostridia	Firmicutes
Soil	Bifidobacteriales	1	Actinobacteria	Actinobacteria
Soil	Chromatiales	1	Gamma Proteobacteria	Proteobacteria
Soil	Flavobacteriales	1	Flavobacteria	Bacteroidetes
Soil	Pseudomonadales	1	Gamma Proteobacteria	Proteobacteria
Soil	Rhizobiales	1	Alpha Proteobacteria	Proteobacteria
Soil	Sphingomonadales	1	Alpha Proteobacteria	Proteobacteria
Soil	Campylobacterales	2	Epsilon Proteobacteria	Proteobacteria

Soil	Coriobacteriales	2	Actinobacteria	Actinobacteria
Soil	Fusobacteriales	2	Fusobacteria	Fusobacteria
Soil	Pasteurellales	2	Gamma Proteobacteria	Proteobacteria
Soil	Selenomonadales	2	Negativicutes	Firmicutes
Soil	Cytophagales	3	Cytophagia	Bacteroidetes
Soil	Enterobacteriales	3	Gamma Proteobacteria	Proteobacteria
Soil	Oscillatoriales	3	Cyanophyceae	Cyanobacteria

These are the modules delineated through our co-occurrence analysis. Numbers represent the module that a specific order belongs to.

Supplemental Table 3. Co-occurrence modules at the family level.

Ecosystem	Family	Module
Apple	Bacillaceae	1
Apple	Flavobacteriaceae	1
Apple	Leuconostocaceae	1
Apple	Micrococcaceae	1
Apple	Nitrosomonadaceae	1
Apple	Peptostreptococcaceae	1
Apple	Prevotellaceae	1
Apple	Solibacteraceae	1
Apple	Spirochaetaceae	1
Apple	Staphylococcaceae	1
Apple	Syntrophomonadaceae	1
Apple	Thermoactinomycetaceae	1
Apple	Thermoleophilaceae	1
Apple	Clostridiaceae	2
Apple	Cytophagaceae	2
Apple	Intrasporangiaceae	2
Apple	Microbacteriaceae	2
Apple	Mycobacteriaceae	2
Apple	Nocardioidaceae	2
Apple	Oxalobacteraceae	2
Apple	Planococcaceae	2
Apple	Propionibacteriaceae	2
Apple	Pseudonocardiaceae	2
Apple	Sanguibacteraceae	2
Apple	Sphingobacteriaceae	2
Apple	Francisellaceae	3
Apple	Ktedonobacteraceae	3
Apple	Moraxellaceae	3

Apple	Nitrospiraceae	3
Apple	Oscillochloridaceae	3
Apple	Parachlamydiaceae	3
Apple	Rhodobacteraceae	3
Apple	Thermomicrobiaceae	3
Apple	Puniceicoccaceae	4
Apple	Verrucomicrobiaceae	4
Apple	Acidimicrobiaceae	5
Apple	Rhodocyclaceae	5
Apple	Rhodothermaceae	5
Apple	Acidithiobacillaceae	6
Apple	Clostridiales.Family.XVII..Incertae.Sedis	6
Apple	Dermabacteraceae	6
Apple	Gemmatimonadaceae	6
Apple	Idiomarinaceae	6
Apple	Ruminococcaceae	6
Apple	Verrucomicrobia.subdivision.3	6
Apple	Acidobacteriaceae	7
Apple	Acidothermaceae	7
Apple	Erythrobacteraceae	7
Apple	Nostocaceae	7
Apple	Streptomycetaceae	7
Apple	Veillonellaceae	7
Apple	Alcaligenaceae	8
Apple	Brevibacteriaceae	8
Apple	Bradyrhizobiaceae	9
Apple	Micromonosporaceae	9
Apple	Promicromonosporaceae	9
Apple	Clostridiales.Family.XI..Incertae.Sedis	10
Apple	Corynebacteriaceae	10
Apple	Deinococcaceae	11
Apple	Methanobacteriaceae	11
Apple	Trueperaceae	11
Apple	Flammeovirgaceae	12
Apple	Paenibacillaceae	12
Apple	Frankiaceae	13
Apple	Thermoanaerobacteraceae	13
Apple	Kineosporiaceae	14
Apple	Lachnospiraceae	14
Apple	Thermaceae	14
Female	Actinomycetaceae	1
Female	Campylobacteraceae	1
Female	Carnobacteriaceae	1
Female	Coriobacteriaceae	1

Female	Fusobacteriaceae	1
Female	Leptotrichiaceae	1
Female	Micrococcaceae	1
Female	Neisseriaceae	1
Female	Pasteurellaceae	1
Female	Streptococcaceae	1
Female	Veillonellaceae	1
Female	Burkholderiaceae	2
Female	Paenibacillaceae	2
Female	Clostridiales.Family.XI..Incertae.Sedis	3
Female	Corynebacteriaceae	3
Female	Cytophagaceae	3
Female	Enterobacteriaceae	3
Female	Mycobacteriaceae	3
Female	Pseudonocardiaceae	3
Female	Comamonadaceae	4
Female	Microbacteriaceae	4
Female	Moraxellaceae	4
Female	Propionibacteriaceae	4
Female	Sphingomonadaceae	4
Female	Staphylococcaceae	4
Female	Streptosporangiaceae	4
Female	Eubacteriaceae	5
Female	Lachnospiraceae	5
Female	Oxalobacteraceae	6
Female	Xanthomonadaceae	6
Female	Bacteroidaceae	7
Female	Clostridiaceae	7
Female	Peptostreptococcaceae	7
Female	Porphyromonadaceae	7
Female	Ruminococcaceae	7
Male	Acetobacteraceae	1
Male	Caulobacteraceae	1
Male	Moraxellaceae	1
Male	Pseudonocardiaceae	1
Male	Sphingomonadaceae	1
Male	Xanthomonadaceae	1
Male	Acidimicrobiaceae	2
Male	Conexibacteraceae	2
Male	Pseudomonadaceae	2
Male	Cellulomonadaceae	3
Male	Intrasporangiaceae	3
Male	Streptomycetaceae	3
Male	Streptosporangiaceae	3

Male	Corynebacteriaceae	4
Male	Mycobacteriaceae	4
Male	Staphylococcaceae	4
Male	Cytophagaceae	5
Male	Microbacteriaceae	5
Male	Micromonosporaceae	5
Male	Nocardiaceae	5
Male	Nocardioidaceae	5
Male	Oxalobacteraceae	5
Male	Rhodobacteraceae	5
Soil	Acetobacteraceae	1
Soil	Alcaligenaceae	1
Soil	Alcanivoracaceae	1
Soil	Bifidobacteriaceae	1
Soil	Burkholderiaceae	1
Soil	Cellulomonadaceae	1
Soil	Comamonadaceae	1
Soil	Cytophagaceae	1
Soil	Desulfohalobiaceae	1
Soil	Desulfomicrobiaceae	1
Soil	Desulfovibrionaceae	1
Soil	Flavobacteriaceae	1
Soil	Gemmatimonadaceae	1
Soil	Halothiobacillaceae	1
Soil	Hyphomonadaceae	1
Soil	Lachnospiraceae	1
Soil	Methylobacteriaceae	1
Soil	Methylococcaceae	1
Soil	Methylophilaceae	1
Soil	Microbacteriaceae	1
Soil	Micrococcaceae	1
Soil	Nannocystaceae	1
Soil	Nitrosomonadaceae	1
Soil	Nocardioidaceae	1
Soil	Nostocaceae	1
Soil	Oceanospirillaceae	1
Soil	Oxalobacteraceae	1
Soil	Pavlovaceae	1
Soil	Peptococcaceae	1
Soil	Peptostreptococcaceae	1
Soil	Phyllobacteriaceae	1
Soil	Planctomycetaceae	1
Soil	Polyangiaceae	1
Soil	Propionibacteriaceae	1

Soil	Pseudomonadaceae	1
Soil	Rhodobacteraceae	1
Soil	Sphingobacteriaceae	1
Soil	Sphingomonadaceae	1
Soil	Thiotrichaceae	1
Soil	Veillonellaceae	1
Soil	Vibrionaceae	1
Soil	Acidimicrobiaceae	2
Soil	Conexibacteraceae	2
Soil	Coxiellaceae	2
Soil	Nocardiaceae	2
Soil	Pseudonocardiaceae	2
Soil	Thermoactinomycetaceae	2
Soil	Acidithiobacillaceae	3
Soil	Chromatiaceae	3
Soil	Enterobacteriaceae	3
Soil	Geobacteraceae	3
Soil	Legionellaceae	3
Soil	Rhizobiaceae	3
Soil	Rhodocyclaceae	3
Soil	Thermoanaerobacteraceae	3
Soil	Verrucomicrobia.subdivision.3	3
Soil	Xanthomonadaceae	3
Soil	Acidobacteriaceae	4
Soil	Alteromonadaceae	4
Soil	Caulobacteraceae	4
Soil	Halomonadaceae	4
Soil	Methylocystaceae	4
Soil	Pasteurellaceae	4
Soil	Rhodospirillaceae	4
Soil	Solibacteraceae	4
Soil	Bacillaceae	5
Soil	Fusobacteriaceae	5
Soil	Micromonosporaceae	5
Soil	Paenibacillaceae	5
Soil	Planococcaceae	5
Soil	Promicromonosporaceae	5
Soil	Beijerinckiaceae	6
Soil	Clostridiaceae	6
Soil	Frankiaceae	6
Soil	Leptospiraceae	6
Soil	Mycobacteriaceae	6
Soil	Segniliparaceae	6
Soil	Streptosporangiaceae	6

Soil	Thermoleophilaceae	6
Soil	Thermomonosporaceae	6
Soil	Bradyrhizobiaceae	7
Soil	Clostridiales.Family.XIV..Incertae.Sedis	8
Soil	Corynebacteriaceae	9
Soil	Erysipelotrichaceae	10
Soil	Funariaceae	11
Soil	Halanaerobiaceae	12
Soil	Cystobacteraceae	13
Soil	Intrasporangiaceae	13
Soil	Rhodobiaceae	14
Soil	Ectothiorhodospiraceae	15
Soil	Myxococcaceae	15
Soil	Rhodothermaceae	15
Soil	Rickettsiaceae	15
Soil	Rivulariaceae	15
Soil	Tsukamurellaceae	15
Soil	Beutenbergiaceae	16
Soil	Hyphomicrobiaceae	16
Soil	Ruminococcaceae	16
Soil	Crenotrichaceae	17
Soil	Nocardiopsaceae	17
Soil	Streptomycetaceae	17
Soil	Victivallaceae	18

These are the modules delineated through our co-occurrence analysis. Numbers represent the module that a specific order belongs to.

Supplemental Table 4. Mixed model results for predicting betweenness from degree.

Taxonomic Level	Ecosystem	Correlation Cutoff	Alpha	SE		Beta	SE	
Family	apple	-0.75						
Family	apple	-0.50	1264263	1.57	***	3.12	0.12	***
Family	apple	0.50	4675	1.27	***	1.57	0.08	***
Family	apple	0.75	36	1.68	***	0.83	0.13	***
Family	female	-0.75	2345	9.78		2.86	0.26	***
Family	female	-0.50	916	3.78	*	2.08	0.07	***
Family	female	0.50	75	1.22	***	0.73	0.10	***
Family	female	0.75	172	1.67	**	1.36	0.08	***
Family	male	-0.75	74	4.53		2.21	0.15	***
Family	male	-0.50	963	1.87	***	1.95	0.07	***

Family	male	0.50	99	1.20	***	0.75	0.06	***
Family	male	0.75	508	1.36	***	1.43	0.08	***
Family	soil	-0.75	13630	7.39		2.30	0.08	***
Family	soil	-0.50	992	5.87		2.02	0.04	***
Family	soil	0.50	122	2.10		0.81	0.12	***
Family	soil	0.75	2208	4.22		1.72	0.17	***
Order	apple	-0.75	7	1.12	**	1.18	0.04	***
Order	apple	-0.50	40538	2.16	***	2.85	0.18	***
Order	apple	0.50	5768	1.63	***	2.00	0.17	***
Order	apple	0.75	8	1.27	***	0.69	0.08	***
Order	female	-0.75	77	1.93	*	1.76	0.13	***
Order	female	-0.50	148	1.79	**	1.76	0.08	***
Order	female	0.50	36	1.95	*	0.92	0.18	***
Order	female	0.75	21	1.39	***	1.08	0.11	***
Order	male	-0.75	6	1.97		1.33	0.07	***
Order	male	-0.50	87	1.97	***	1.91	0.08	***
Order	male	0.50	51	1.28	***	1.01	0.12	***
Order	male	0.75	52	1.32	***	1.21	0.10	***
Order	soil	-0.75	584	1.54	**	1.72	0.10	***
Order	soil	-0.50	1224	3.39		1.91	0.08	***
Order	soil	0.50	132	1.90	*	0.93	0.19	***
Order	soil	0.75	441	1.28		1.44	0.09	

In the table, SE represents the standard error of each parameter. Asterisks represent p-values for each parameter where *** represents <0.001, ** represents <0.01, and * represents <0.05. Missing parameter values represent cases where sufficient data points were not available.

Supplemental Table 5. Clustering Coefficient Statistics

Taxonomic Level	Ecosystem	Correlation Cutoff	Clustering Coefficient	SE	Random Coefficient	SE	Clustering Ratio	SE
Order	Apple	-0.75	0.00	0.00	0.54	0.14		
	Female	-0.75	0.00	0.00	0.04	0.01		
	Male	-0.75	0.00	0.00	0.00	0.00		
	Soil	-0.75	0.00	0.00	0.12	0.00	0.00	0.00
	Apple	-0.5	0.00	0.00	0.10	0.00	0.00	0.00
	Female	-0.5	0.00	0.00	0.19	0.00	0.00	0.00
	Male	-0.5	0.00	0.00	0.13	0.01		
	Soil	-0.5	0.00	0.00	0.16	0.00	0.00	0.00
	Apple	-0.25	0.04	0.00	0.15	0.00	0.24	0.01
	Female	-0.25	0.06	0.00	0.24	0.01	0.24	0.01
	Male	-0.25	0.06	0.00	0.16	0.00	0.41	0.02
	Soil	-0.25	0.09	0.00	0.30	0.01	0.30	0.00
	Apple	0.25	0.52	0.00	0.27	0.00	1.93	0.01

	Female	0.25	0.78	0.01	0.46	0.01	1.76	0.02
	Male	0.25	0.76	0.00	0.47	0.00	1.62	0.01
	Soil	0.25	0.63	0.01	0.38	0.01	1.66	0.00
	Apple	0.5	0.45	0.00	0.07	0.00	6.76	0.21
	Female	0.5	0.73	0.01	0.28	0.01	2.99	0.11
	Male	0.5	0.67	0.00	0.28	0.00	2.52	0.04
	Soil	0.5	0.60	0.01	0.21	0.01	2.93	0.04
	Apple	0.75	0.61	0.01	0.00	0.00		
	Female	0.75	0.72	0.01	0.22	0.01	3.92	0.25
	Male	0.75	0.61	0.00	0.16	0.00	3.94	0.06
	Soil	0.75	0.54	0.01	0.12	0.01	9.81	0.63
Family	Apple	-0.75	0.00	0.00	0.00	0.00		
	Female	-0.75	0.00	0.00	0.05	0.02		
	Male	-0.75	0.00	0.00	0.17	0.03		
	Soil	-0.75	0.00	0.00	0.13	0.01	0.00	0.00
	Apple	-0.5	0.00	0.00	0.04	0.00	0.00	0.00
	Female	-0.5	0.00	0.00	0.07	0.00		
	Male	-0.5	0.00	0.00	0.06	0.00		
	Soil	-0.5	0.04	0.00	0.23	0.01	0.11	0.01
	Apple	-0.25	0.04	0.00	0.12	0.00	0.32	0.00
	Female	-0.25	0.06	0.00	0.15	0.00	0.45	0.01
	Male	-0.25	0.05	0.00	0.13	0.00	0.41	0.01
	Soil	-0.25	0.11	0.00	0.33	0.00	0.35	0.01
	Apple	0.25	0.54	0.00	0.30	0.00	1.78	0.00
	Female	0.25	0.78	0.00	0.48	0.01	1.76	0.03
	Male	0.25	0.75	0.00	0.48	0.00	1.56	0.01
	Soil	0.25	0.72	0.01	0.42	0.00	1.71	0.01
	Apple	0.5	0.37	0.00	0.08	0.00	4.64	0.03
	Female	0.5	0.73	0.00	0.29	0.00	2.70	0.06
	Male	0.5	0.65	0.00	0.27	0.00	2.49	0.01
	Soil	0.5	0.68	0.01	0.31	0.01	2.39	0.03
	Apple	0.75	0.66	0.00	0.02	0.00		
	Female	0.75	0.65	0.00	0.13	0.00	5.48	0.09
	Male	0.75	0.61	0.00	0.08	0.00	7.96	0.10
	Soil	0.75	0.69	0.01	0.16	0.01	6.92	0.22

In the table, SE represents the standard error of each parameter.

Supplemental Table 6. Node labels for Figure 3.

Microbial Order	Nodel Label	Microbial Order	Nodel Label
Acidimicrobiales	1	Halanaerobiales	34
Acidobacteriales	2	Methylococcales	35
Alteromonadales	3	Methylophilales	36
Burkholderiales	4	Neisseriales	37
Caulobacterales	5	Nitrosomonadales	38
Cytophagales	6	Pasteurellales	39
Desulfovibrionales	7	Pseudomonadales	40
Flavobacteriales	8	Rhodocyclales	41
Gemmatimonadales	9	Rickettsiales	42
Legionellales	10	Selenomonadales	43
Myxococcales	11	Synergistales	44
Nostocales	12	Thermoanaerobacterales	45
Oceanospirillales	13	Thermoleophilales	46
Prochlorales	14	Verrucomicrobiales	47
Rhodobacterales	15	Vibrionales	48
Solibacterales	16	Xanthomonadales	49
Solirubrobacterales	17	Puniceicoccales	50
Sphingobacteriales	18	Chlamydiales	51
Sphingomonadales	19	Chlorobiales	52
Thiotrichales	20	Chloroflexales	53
Trichomonadida	21	Clostridiales	54
Acidithiobacillales	22	Ktedonobacterales	55
Actinomycetales	23	Lactobacillales	56
Bacillales	24	Rhodospirillales	57
Bacteroidales	25	Thermomicrobiales	58
Bacteroidetes Order II Incertae sedis	26	Bifidobacteriales	59
Bdellovibrionales	27	Campylobacterales	60
Chromatiales	28	Coriobacteriales	61
Chroococcales	29	Cytophagales	62
Desulfurellales	30	Flavobacteriales	63
Desulfuromonadales	31	Oscillatoriales	64
Enterobacteriales	32	Rhizobiales	65
Fusobacteriales	33		

Numbers represent the node label for Figure 2.

2.2 Figures

Figure 1. Community NMDS plots

The NMDS plot shows differences in community composition between ecosystems at two different levels of taxonomy. The plot on the left (A) shows differences among microbial orders while differences on the right (B) represent differences in microbial families. Each point represents a sample within an ecosystem, and ellipses represent the standard deviation around a centroid for each ecosystem.

Figure 2. Networks of negative co-occurring microbial orders within ecosystems

Networks represent relationships between co-occurring ecosystems. Edges colored in black represent co-occurrence relationships that were consistent at the -0.75 correlation level, while edges in grey represent co-occurrence relationships that were consistent at the -0.5 correlation level. Numbers represent microbial orders seen in the table.

Figure 3. Power-function relationships between node degree and betweenness across all correlation levels

Figures represent the power-function relationships between node degree and betweenness for microbial orders and families within each ecosystem at all correlation levels. Scales are log transformed. Each best-fit line represents the predicted values seen in Supplementary Table 4 for each correlation cutoff.

Figure 1.

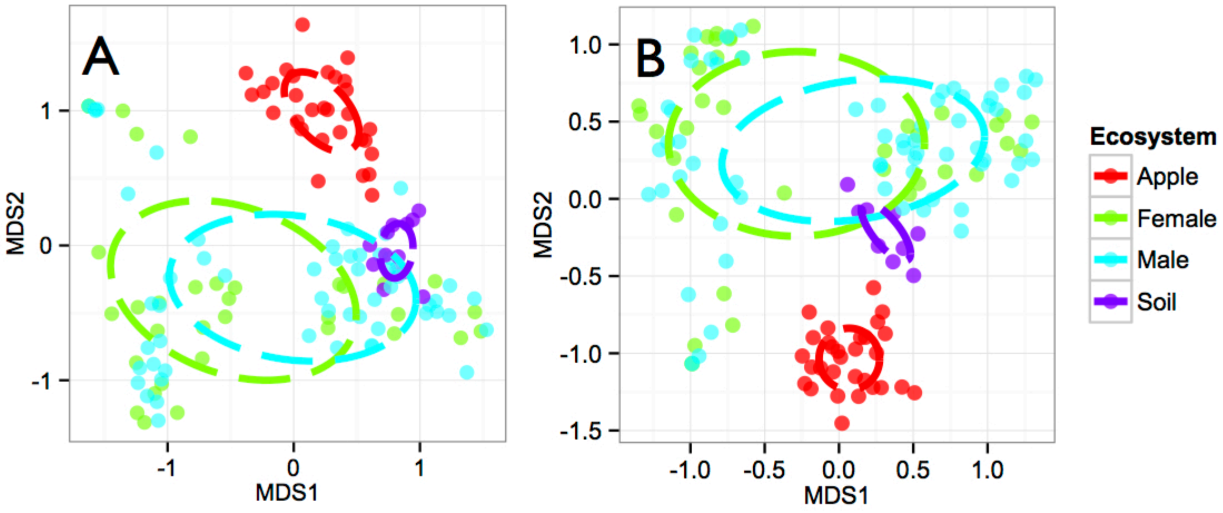
A
B
MDS1
MDS2
-1
0
1
1.0
0.5
0.0
-0.5
-1.0
-1.5
Ecosystem
Apple
Female
Male
Soil

Figure 2.

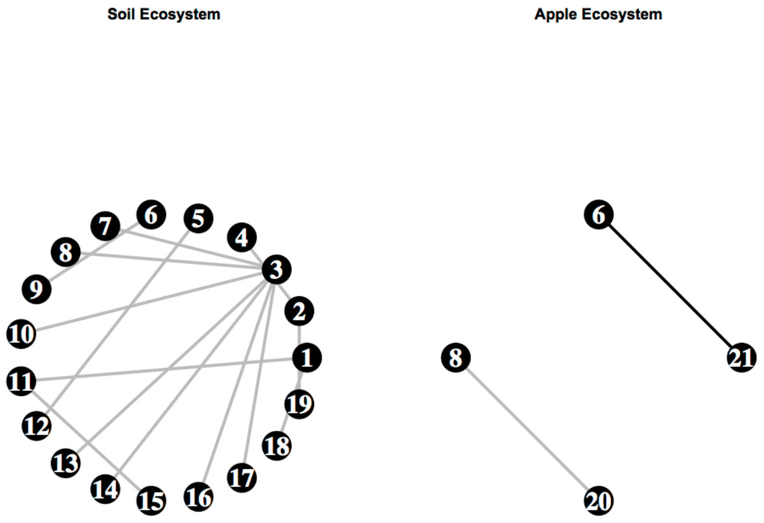

Orders	Number
Acidimicrobiales	1
Acidithiobacillales	2
Acidobacteriales	3
Actinomycetales	4
Bacteroidales	5
Enterobacteriales	6
Erysipelotrichales	7
Flavobacteriales	8
Lactobacillales	9
Methylococcales	10
Oscillatoriales	11
Rhizobiales	12
Rhodobacterales	13
Selenomonadales	14
Solirubrobacterales	15
Sphingobacteriales	16
Sphingomonadales	17
Thermoanaerobacterales	18
Thiotrichales	19
Nitrospirales	20
Deinococcales	21

Figure 3.

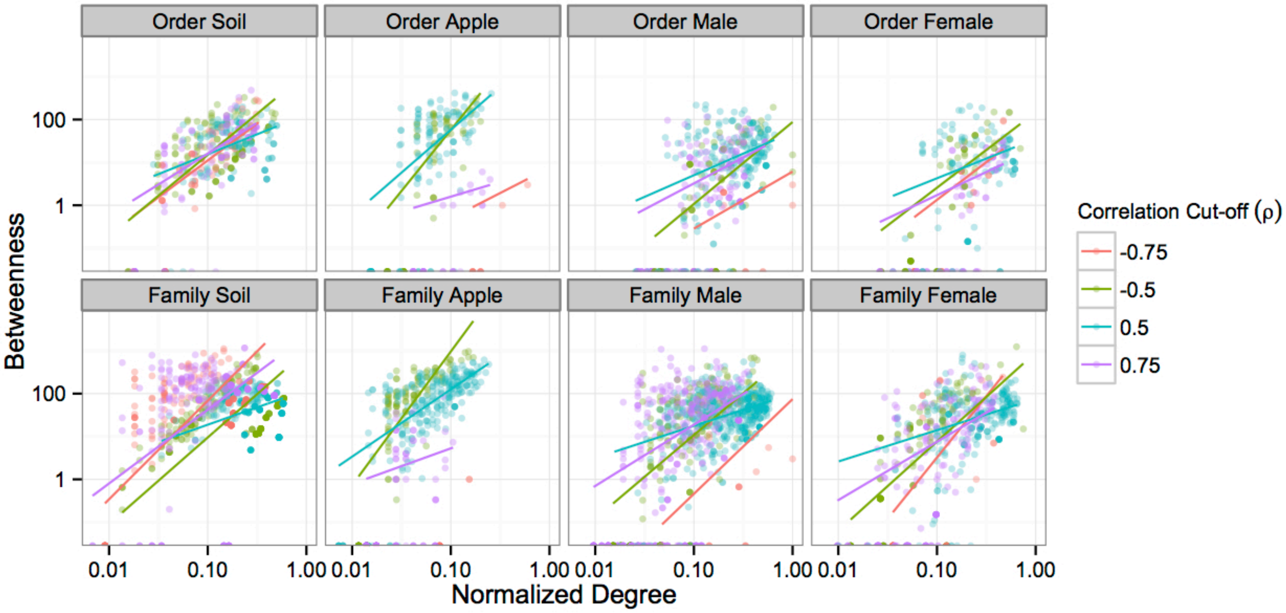
Order Soil
Order Apple
Order Male
Order Female
Family Soil
Family Apple
Family Male
Family Female
Betweenness
100
1
0.01
0.10
1.00
Normalized Degree
Correlation Cut-off (ρ)
-0.75
-0.5
0.5
0.75
